# TRIM28-mediated SUMOylation of SREBF1 drives PD‑L1 N‑glycosylation and immune evasion in bladder cancer

**DOI:** 10.1038/s41419-026-08798-8

**Published:** 2026-05-18

**Authors:** Ru Chen, Yuzhe Su, Jianhui Chen, Shaoxing Zhu, Xiaobao Chen, Yiming Su

**Affiliations:** Department of Urology, Fujian Medical University Union Hospital, Fuzhou, PR China

**Keywords:** Tumour immunology, Urological cancer

## Abstract

Bladder cancer progression is frequently accompanied by metabolic reprogramming and immune escape, yet how these processes are coupled remains unclear. Sterol regulatory element-binding transcription factor 1 (SREBF1) governs lipid homeostasis and is often deregulated in cancer. We find that SREBF1 is upregulated in human bladder tumours and associates with invasion, metastasis and poor patient outcome, critically, it sustains tumor growth by driving immune evasion. Mechanistically, SREBF1 directly interacts with the SUMO E3 ligase TRIM28, which catalyses SUMO2 conjugation of SREBF1 at K470, antagonizes K48-linked ubiquitination and stabilizes SREBF1. TRIM28 further enhances SREBF1 occupancy at the MGAT4A promoter and upregulates MGAT4A, thereby promoting N-linked glycosylation of PD-L1, which stabilizes PD-L1 and increases its plasma-membrane localization, dampening CD8 + T-cell cytotoxicity. In immunocompetent mice, TRIM28 knockdown reduces tumor growth, lowers PD-L1 and increases CD8 + T-cell infiltration—effects reversed by SREBF1 overexpression; in an immune-augmented human PDX model, SREBF1 targeting synergizes with anti–PD-1 without overt toxicity. These findings identify a TRIM28–SREBF1–MGAT4A axis that couples lipid metabolic rewiring to immune-checkpoint regulation via PD-L1 N-glycosylation, positioning SREBF1 as a biomarker for BCa progression and risk stratification and as a druggable node to potentiate PD-1 blockade.

## Introduction

Bladder cancer (BCa) is among the most prevalent and lethal malignancies of the urinary tract worldwide [1–3]. Approximately three quarters of patients present with non-muscle-invasive disease (NMIBC), with the remainder diagnosed as muscle-invasive (MIBC) [4]. Despite advances in perioperative multimodal therapy and platinum-based chemotherapy, the natural history of NMIBC remains characterized by frequent recurrence and that of MIBC by a high propensity for metastasis [5, 6]. Immune checkpoint blockade targeting PD-1/PD-L1 has delivered durable benefit to a subset of patients; however, objective response rates are modest and both primary and acquired resistance are common [7, 8]. These observations underscore that key molecular determinants and pathways of tumor immune evasion remain incompletely defined, particularly the protein quality-control and post-translational modification networks that govern PD-L1 homeostasis and membrane residency in BCa.

Metabolic reprogramming is a central hallmark of cancer, with lipid metabolism orchestrating membrane biogenesis, signal transduction, redox balance, and epigenetic and immune regulation. Sterol regulatory element-binding protein 1 (SREBF1) is a master transcriptional regulator of fatty acid and cholesterol biosynthetic genes and has been implicated across cancers in invasion, metastasis, therapeutic resistance, and remodeling of the tumor immune microenvironment [9, 10]. Yet, in BCa, the clinical relevance of SREBF1, its potential to drive immune escape through noncanonical mechanisms beyond lipid metabolites, and the putative “metabolism-immunity” coupling that links SREBF1 to immune regulation remain poorly defined [11–13]. Post-translational modifications (PTMs) integrate cellular stress and signaling networks to fine-tune protein fate. SUMOylation frequently interacts with and counterbalances ubiquitination, thereby modulating substrate stability, subcellular localization, and transcriptional programs [14, 15]. TRIM family proteins function as scaffolds and E3 ligases in PTM regulation; among them, TRIM28 (also known as KAP1) exerts pleiotropic roles in tumorigenesis, stress responses, and chromatin regulation, but its substrate spectrum and immune-related functions in BCa are not well understood [16–18]. In parallel, N-linked glycosylation of PD-L1 is known to enhance its stability, promote membrane localization, and blunt T-cell cytotoxicity, with branched N-glycan formation dependent on MGAT family N-acetylglucosaminyltransferases [19, 20]. How metabolic transcriptional programs such as SREBF1 shape the expression of glycosyltransferases to dictate PD-L1 fate represents a critical yet insufficiently defined axis linking metabolism to immune escape.

Against this backdrop, we integrate multi-cohort transcriptomic and pathological analyses with cell biology, proteomic/biochemical mapping of protein interactions and modifications, glycomics-focused interrogation, immune co-culture assays, and studies in immunocompetent mouse models and an immune-augmented patient-derived xenograft (PDX) system. We define the expression landscape and prognostic association of SREBF1 in BCa; establish TRIM28 as an E3 SUMO ligase that directly modifies and stabilizes SREBF1; and demonstrate their cooperative regulation of MGAT4A at the chromatin level, which in turn drives PD-L1 N-glycosylation, stabilization, and membrane localization, with functional consequences for CD8 + T-cell effector activity and tumor growth. Building on these mechanistic insights, we evaluate the therapeutic feasibility of combining SREBF1 targeting with anti-PD-1, providing proof-of-concept for metabolic-immune pathway-based molecular stratification and precision combination therapy. Collectively, this work delineates a pathway that links a lipogenic master regulator to protein quality control, glycosylation, and immune evasion, offering a molecular framework and actionable targets for risk stratification and optimization of immunotherapy in BCa.

## Results

### SREBF1 overexpression correlates with tumor progression and poor prognosis in BCa

**Fig. 1:**
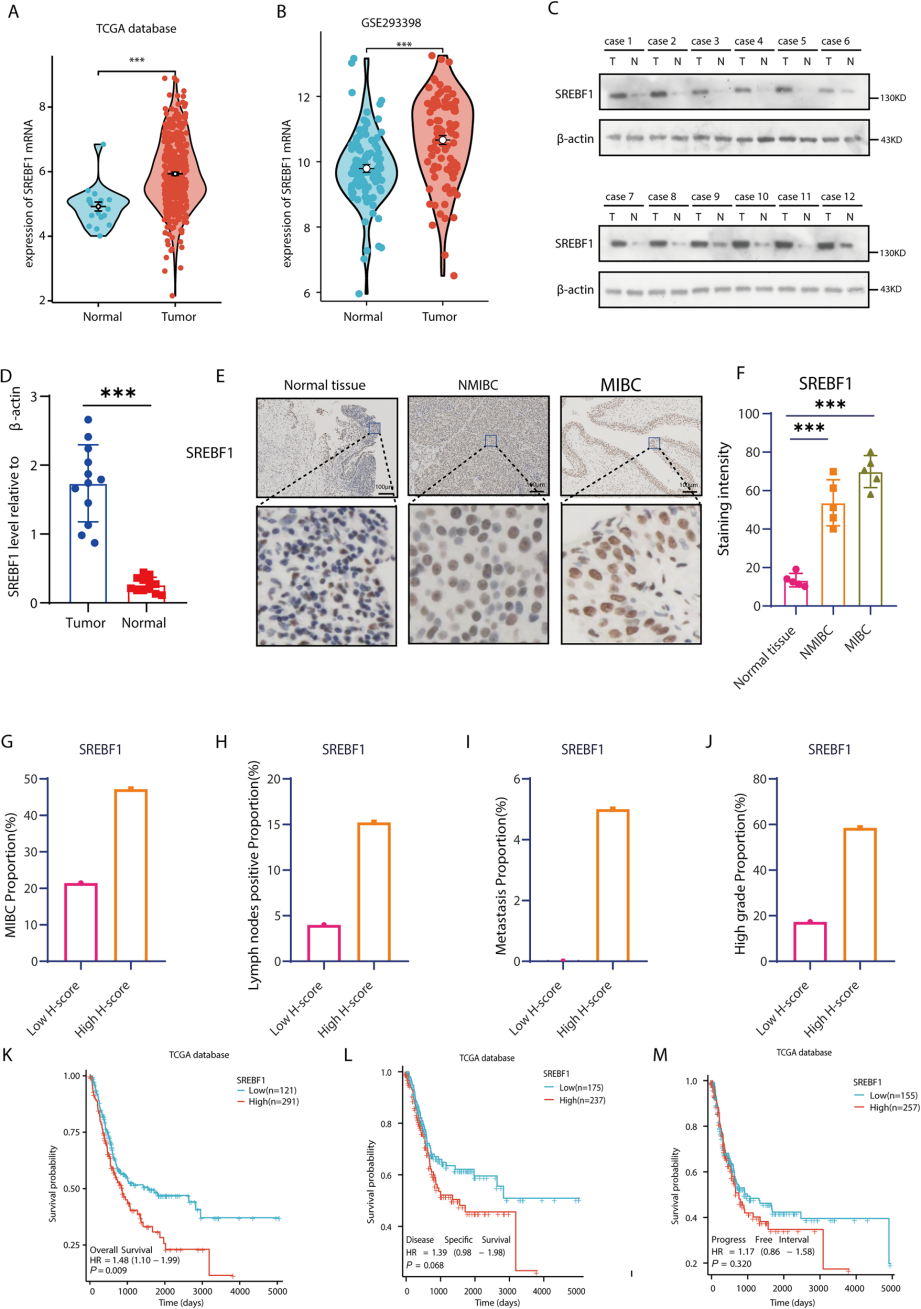
SREBF1 is overexpressed in bladder cancer and associates with aggressive disease and poor prognosis. **A**, **B** SREBF1 mRNA abundance in tumour versus normal bladder tissues across TCGA-BLCA and GSE293398 cohorts. **C**, **D** Immunoblot of SREBF1 in 12 paired BCa and adjacent normal tissues with quantitative densitometry (normalized to loading control). **E** Representative IHC staining of SREBF1 in tumour and adjacent nonmalignant tissues and quantification of SREBF1-positive cases in 30 patients. **F** Increased frequency of SREBF1 positivity in muscle-invasive bladder cancer (MIBC) relative to non–muscle-invasive disease (NMIBC). **G**–**J** Clinicopathological correlations showing higher SREBF1 expression in cases with muscle invasion, lymph-node metastasis, distant dissemination and higher tumour grade. **K**–**M** Kaplan–Meier analyses (TCGA-BLCA) demonstrate worse overall survival, cancer-specific survival and progression-free interval in SREBF1-high versus SREBF1-low groups. Data are presented as indicated; box plots show median and IQR with whiskers to 1.5× IQR, unless otherwise stated. Two-sided statistical tests were used; survival differences were assessed by log-rank test. *P* values: ns, not significant; *, *P* < 0.05; **, *P* < 0.01; ***, *P* < 0.001.

SREBF1, a master transcription factor governing lipid metabolism, has been increasingly recognized for its role in tumorigenesis [21]. To elucidate its relevance in bladder cancer, we analyzed transcriptional profiles from The Cancer Genome Atlas (TCGA) and Gene Expression Omnibus (GSE293398). In both independent cohorts, SREBF1 mRNA levels were markedly upregulated in tumor tissues compared with normal bladder tissues (Fig. 1A and B). At the protein level, immunoblot analysis of 12 paired BCa and adjacent normal tissues revealed a consistent elevation of SREBF1 abundance in tumors (Fig. 1C and D). Furthermore, immunohistochemical (IHC) staining of pathological specimens from 30 patients demonstrated a significantly higher proportion of SREBF1-positive cases in tumor tissues relative to adjacent nonmalignant tissues (Fig. 1E). Notably, SREBF1 positivity was more frequent in muscle-invasive bladder cancer (MIBC) than in non-muscle-invasive bladder cancer (NMIBC) (Fig. 1F). Clinicopathological correlation analyses showed that high SREBF1 expression was significantly associated with muscle invasion, lymph node metastasis, distant dissemination, and higher tumor grade (Fig. 1G-J). Survival analyses using TCGA data further indicated that patients with elevated SREBF1 expression exhibited poorer overall survival (OS), cancer-specific survival (CSS), and progression-free interval (PFI) than those with low expression(Fig. 1K-M). Collectively, these multi-cohort and multi-level findings demonstrate that SREBF1 is consistently overexpressed in BCa and closely associated with tumor progression and unfavorable prognosis, suggesting its potential utility as a molecular indicator of disease advancement and risk stratification.

### SREBF1 silencing reduces BCa cell migration and invasion

**Fig. 2:**
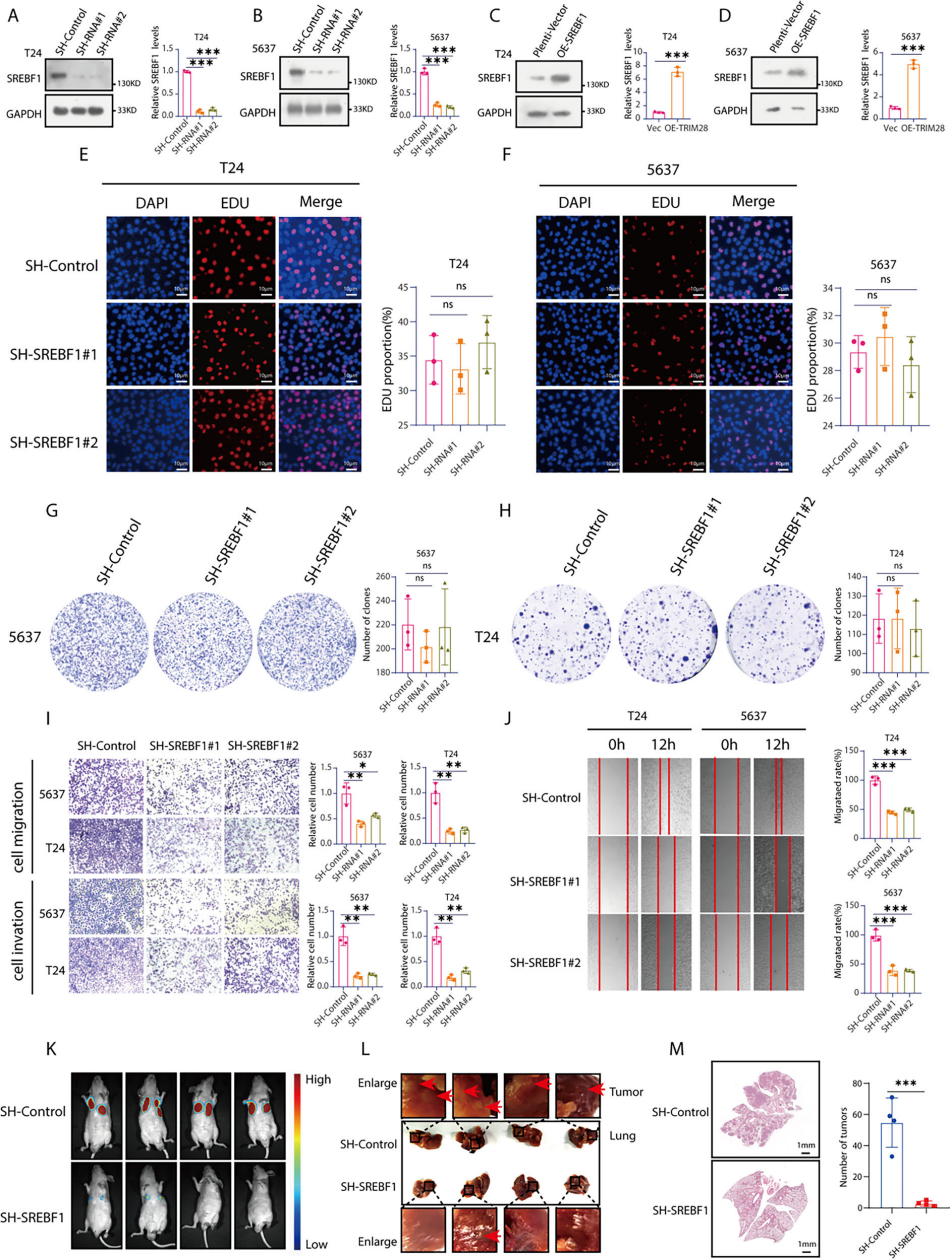
SREBF1 promotes migration, invasion and lung colonization of bladder cancer cells without altering proliferation. **A**–**D** Validation of SREBF1 knockdown (shRNA) and overexpression in T24 and 5637 cells. **E**–**H** Proliferation is unchanged upon SREBF1 silencing, as assessed by EdU incorporation and colony-formation assays. **I** Transwell migration and invasion are markedly reduced by SREBF1 knockdown in both cell lines. **J** Wound-healing assays independently confirm impaired migration following SREBF1 downregulation. **K**–**M** In vivo metastasis model: tail-vein injection of luciferase-labelled T24 cells into BALB/c nude mice shows that SREBF1 knockdown decreases pulmonary metastatic burden (bioluminescence imaging) and reduces lung nodule counts. Data are mean ± s.d. unless indicated; two-sided statistical tests were used. *P* values: ns, not significant; *, *P* < 0.05; **, *P* < 0.01; ***, *P* < 0.001.

To define the functional contribution of SREBF1 in BCa, we generated shRNA-mediated knockdown vectors and an SREBF1 overexpression plasmid and confirmed modulation efficiency in the T24 and 5637 cell lines (Fig. 2A-D). Functionally, SREBF1 silencing did not significantly alter proliferation, as measured by EdU incorporation, a result corroborated by colony-formation assays (Fig. 2E-H). In contrast, Transwell assays showed that SREBF1 knockdown markedly impaired the migratory and invasive capacities of both cell lines (Fig. 2I), which was independently supported by wound-healing assays demonstrating reduced migration upon SREBF1 downregulation (Fig. 2J). In an in vivo metastasis model, tail-vein injection of luciferase-labelled T24 cells into BALB/c nude mice revealed that SREBF1 knockdown significantly reduced pulmonary metastatic burden and tumour nodule formation (Fig. 2K-M). Collectively, these in vitro and in vivo data indicate that, while SREBF1 does not measurably influence proliferative growth, it promotes migration, invasion and lung colonization of BCa cells, implicating SREBF1 in metastatic progression.

### SREBF1 interacts with TRIM28

**Fig. 3:**
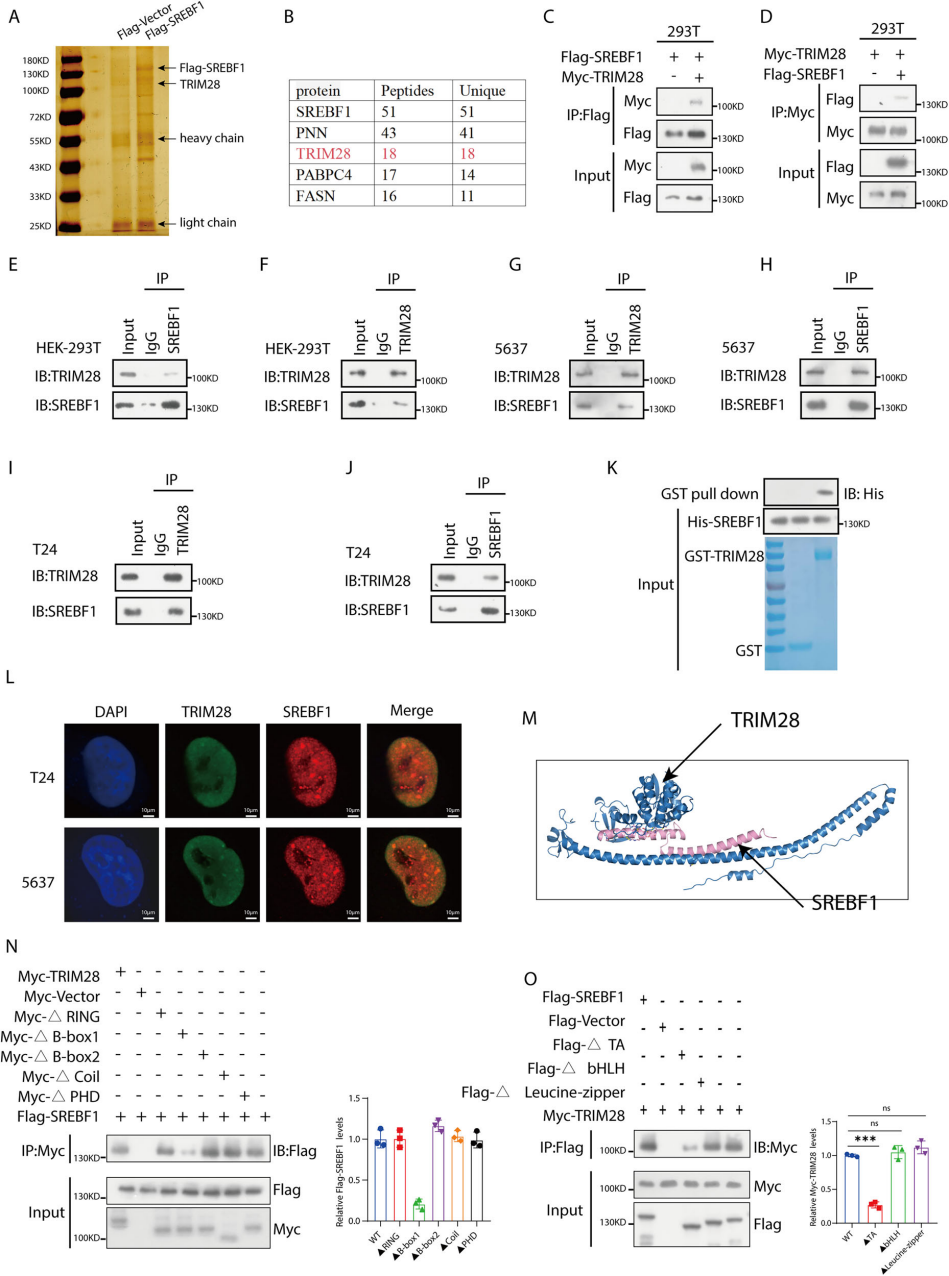
TRIM28 directly interacts with SREBF1 and co-localizes in the nucleus; the TRIM28 B-box1 and SREBF1 TA domains mediate binding. **A** Co-immunoprecipitation (co-IP) of Flag–SREBF1 from T24 cells followed by silver staining reveals distinct protein bands in the precipitated complex. **B** LC–MS/MS of the Flag–SREBF1 co-IP identifies TRIM28 as a significantly enriched interactor. **C**, **D** Reciprocal co-IP in HEK293T cells stably expressing Flag–SREBF1 and Myc–TRIM28 using anti-Flag or anti-Myc antibodies confirms a robust physical interaction. **E**–**J** Endogenous co-IP with anti-TRIM28 or anti-SREBF1 antibodies in HEK293T, T24 and 5637 cells corroborates the interaction under physiological expression. **K** In vitro GST pull-down using recombinant GST–TRIM28 demonstrates direct binding to SREBF1. **L** Immunofluorescence microscopy shows nuclear co-localization of TRIM28 and SREBF1 in T24 and 5637 cells. **M** Molecular docking predicts a stable binding interface between TRIM28 and SREBF1. **N**–**O** Domain mapping with truncation mutants indicates that the TRIM28 B-box1 domain interacts with the SREBF1 transcriptional activation (TA) domain, as shown by co-IP in HEK293T cells. Inputs and IP fractions are shown where indicated.

To elucidate the molecular mechanism by which TRIM28 functions in BCa, we sought to identify its potential protein interaction partners. We ectopically expressed Flag-tagged SREBF1 in T24 cells and performed co-immunoprecipitation (co-IP) using anti-Flag magnetic beads, followed by silver staining. Distinct protein bands were observed in the immunoprecipitated complex, indicative of specific interacting proteins (Fig. 3A). Liquid chromatography-tandem mass spectrometry (LC–MS/MS) identified TRIM28 as a significantly enriched interactor (Fig. 3B), suggesting a functional association between TRIM28 and SREBF1. To validate this interaction, we established HEK293T cell lines stably expressing Flag-tagged SREBF1 and Myc-tagged TRIM28. Reciprocal co-IP using anti-Flag and anti-Myc antibodies confirmed a robust physical interaction between TRIM28 and SREBF1 (Fig. 3C and D). Moreover, endogenous co-IP with anti-TRIM28 and anti-SREBF1 antibodies in HEK293T, T24, and 5637 cells further corroborated the interaction under physiological expression conditions (Fig. 3E–J). To determine whether TRIM28 directly binds SREBF1, we performed in vitro GST pull-down assays using recombinant GST-tagged TRIM28. These experiments demonstrated that TRIM28 directly interacts with SREBF1 in a cell-free system (Fig. 3K). Consistently, immunofluorescence microscopy revealed nuclear co-localization of TRIM28 and SREBF1 in T24 and 5637 cells, implying potential cooperation in nuclear processes (Fig. 3L). In addition, molecular docking simulations predicted a stable binding interface between TRIM28 and SREBF1, providing structural support for a direct interaction (Fig. 3M). To map the interaction domains, we generated a series of truncation mutants of TRIM28 and SREBF1 based on their known domain architectures. Co-IP assays in HEK293T cells expressing the respective mutants revealed that the B-box1 domain of TRIM28 mediates its interaction with the transcriptional activation (TA) domain of SREBF1 (Fig. 3N and O). Collectively, these findings establish a direct, domain-specific interaction between TRIM28 and SREBF1, suggesting that TRIM28 may modulate SREBF1 function through physical association in the nucleus.

### TRIM28 acts as an E3 SUMO ligase for SREBF1 and regulates its SUMOylation in BCa

**Fig. 4:**
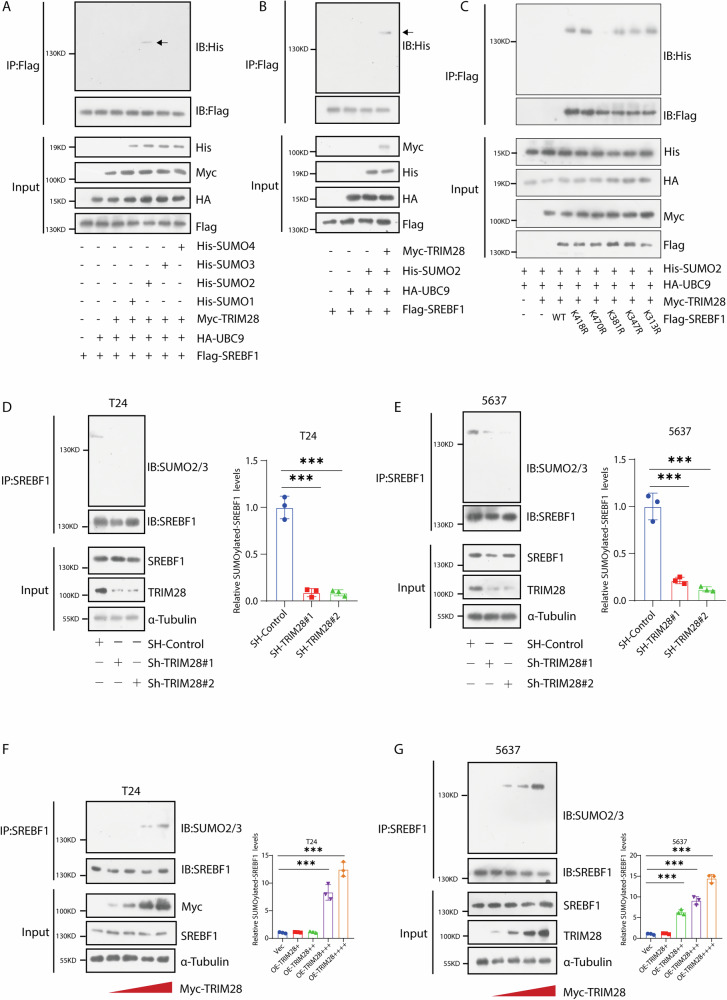
TRIM28 functions as an E3 SUMO ligase for SREBF1 and mediates SUMO2 conjugation at K470. **A**, **B** HEK293T cells co-expressing Flag–SREBF1, Myc–TRIM28, HA–UBC9 and His-tagged SUMO isoforms (SUMO1–4) were subjected to anti-Flag immunoprecipitation (IP); TRIM28 selectively enhances SUMO2 modification of SREBF1 (quantification in **B**). **C** Site mapping by K → R mutagenesis of GPS-SUMO–predicted lysines identifies K470 as the major SUMOylation site, as K470R markedly reduces SUMOylation. **D**, **E** TRIM28 knockdown decreases endogenous SREBF1 SUMOylation in T24 and 5637 bladder cancer cells. **F**, **G** TRIM28 overexpression increases SREBF1 SUMO2 conjugation in a dose-dependent manner. SUMOylated SREBF1 was detected in anti-Flag IPs; inputs and IP fractions are shown where indicated. Data are mean ± s.d. where quantified; two-sided tests were used. *P* values: ns, not significant; *, *P* < 0.05; **, *P* < 0.01; ***, *P* < 0.001.

Post-translational SUMOylation plays a pivotal role in regulating the function of cancer-associated proteins [22, 23]. To investigate whether TRIM28 promotes the SUMOylation of SREBF1 in BCa, we co-transfected HEK293T cells with His-tagged SUMO isoforms (SUMO1-4), Flag-tagged SREBF1, Myc-tagged TRIM28, and HA-tagged UBC9, followed by immunoprecipitation using anti-Flag magnetic beads. The results revealed that TRIM28 specifically enhanced SUMO2 modification of SREBF1 (Fig. 4Aand B). To identify potential SUMOylation sites on SREBF1, we utilized the GPS-SUMO prediction algorithm (Figure S1) and selected five candidate lysine residues for site-directed mutagenesis (K → R). Among these, mutation at K470 markedly reduced the SUMOylation level of SREBF1 (Fig. 4C), indicating that K470 serves as the major SUMOylation site on SREBF1. Consistently, knockdown of TRIM28 in T24 and 5637 BCa cell lines significantly decreased SREBF1 SUMOylation (Fig. 4D and E), whereas TRIM28 overexpression led to a dose-dependent increase in SUMO2 modification of SREBF1 (Fig. 4F and G). These findings collectively demonstrate that TRIM28 functions as an E3 SUMO ligase that specifically mediates SUMO2 conjugation of SREBF1 at lysine 470, thereby potentially regulating its activity in BCa cells.

### TRIM28 enhances SREBF1 stability through SUMOylation-dependent inhibition of K48-linked ubiquitination

**Fig. 5:**
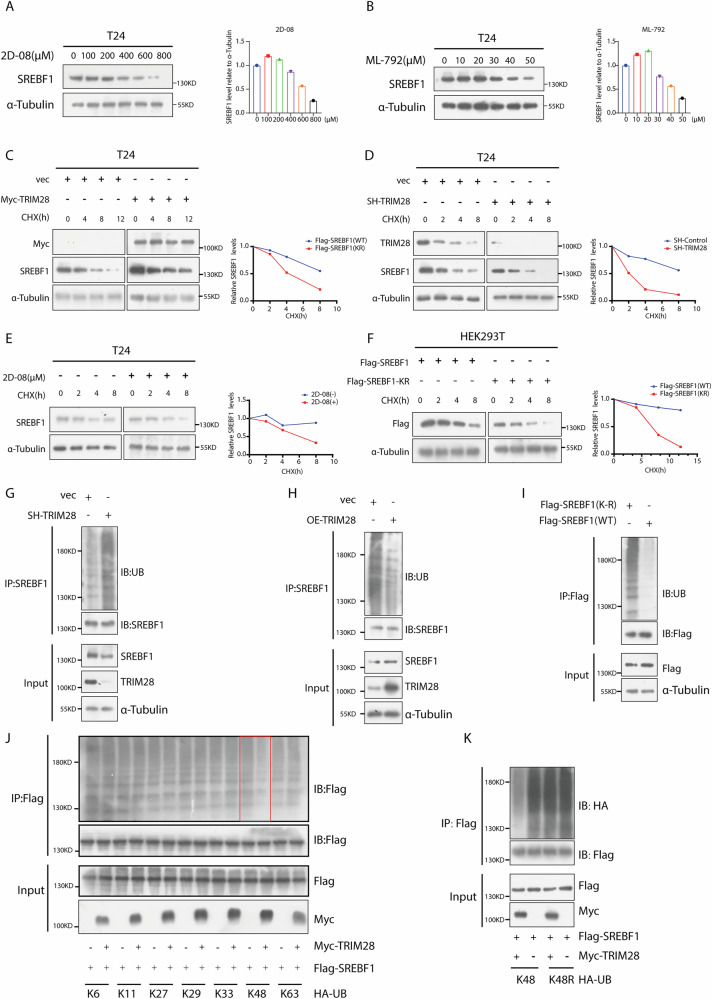
TRIM28-mediated SUMOylation stabilizes SREBF1 by antagonizing K48-linked ubiquitination. **A**, **B** SUMOylation inhibitors 2-D08 and ML-792 reduce SREBF1 protein levels in T24 cells in a dose-dependent manner. **C**, **D** Cycloheximide (CHX) chase assays show that TRIM28 knockdown accelerates, whereas TRIM28 overexpression prolongs, SREBF1 half-life. **E** Inhibition of SUMOylation by 2-D08 accelerates SREBF1 degradation. **F** The non-SUMOylatable mutant SREBF1^KR displays faster degradation than wild-type SREBF1 in CHX chase assays. **G**, **H** Endogenous ubiquitination analysis reveals that TRIM28 depletion increases, while TRIM28 overexpression decreases, SREBF1 ubiquitination. **I** Ubiquitination of SREBF1^KR is elevated compared with SREBF1^WT. **J** Co-transfection with lysine-specific ubiquitin mutants demonstrates that TRIM28 selectively suppresses K48-linked ubiquitination of SREBF1. **K** Overexpression of the ubiquitin K48R mutant abolishes the inhibitory effect of TRIM28 on SREBF1 ubiquitination. Collectively, these data indicate that TRIM28-mediated SUMOylation stabilizes SREBF1 by preventing K48-linked polyubiquitination and proteasomal degradation. Data are mean ± s.d. where applicable; two-sided statistical tests were used. P values: ns, not significant; *, *P* < 0.05; **, *P* < 0.01; ***, *P* < 0.001.

Since numerous studies have shown that SUMOylation often contributes to protein stabilization, we hypothesized that TRIM28-mediated SUMOylation might play a similar role in regulating SREBF1. Treatment of T24 cells with increasing concentrations of the SUMOylation inhibitors 2-D08 and ML-792 led to a marked, dose-dependent reduction in SREBF1 protein levels (Fig. 5A and B), supporting this hypothesis. To further investigate the effect of TRIM28 on SREBF1, we performed cycloheximide (CHX) chase assays. Knockdown of TRIM28 accelerated SREBF1 degradation (Fig. 5D), whereas overexpression of TRIM28 significantly prolonged the half-life of SREBF1 (Fig. 5C). Consistently, treatment of T24 cells with 2-D08 also accelerated SREBF1 degradation (Fig. 5E). Moreover, when a non-SUMOylatable SREBF1 mutant (SREBF1^KR) was overexpressed in 293 T cells, CHX chase assays revealed that the mutant underwent faster degradation compared with wild-type SREBF1 (Fig. 5F), indicating that SUMOylation contributes to the stabilization of SREBF1. Previous studies have suggested that SUMOylation and ubiquitination often compete with each other, thereby influencing protein stability. To determine whether TRIM28 affects the ubiquitination of SREBF1, we examined endogenous SREBF1 ubiquitination levels following TRIM28 knockdown. Loss of TRIM28 markedly increased SREBF1 ubiquitination (Fig. 5G). Conversely, overexpression of TRIM28^WT significantly reduced SREBF1 ubiquitination (Fig. 5H). Similarly, ubiquitination assays in 293 T cells co-transfected with HA-Ub and either SREBF1^WT or SREBF1^KR demonstrated that the SREBF1^KR mutant exhibited higher ubiquitination levels (Fig. 5I). To delineate the type of ubiquitin linkage regulated by TRIM28, we co-transfected 293 T cells with TRIM28 and a panel of lysine-specific ubiquitin mutants (HA-Ub-K6, K11, K27, K33, K48, and K63). TRIM28 specifically suppressed K48-linked ubiquitination of SREBF1 (Fig. 5J). Furthermore, overexpression of the K48R ubiquitin mutant abolished the ability of TRIM28 to reduce SREBF1 ubiquitination, confirming that TRIM28 specifically regulates K48-linked ubiquitin chains (Fig. 5K). Collectively, these findings demonstrate that TRIM28-mediated SUMOylation stabilizes SREBF1 by antagonizing K48-linked ubiquitination, thereby protecting SREBF1 from proteasomal degradation.

### TRIM28-SREBF1 drives MGAT4A-dependent PD-L1 N-glycosylation and immune evasion

**Fig. 6:**
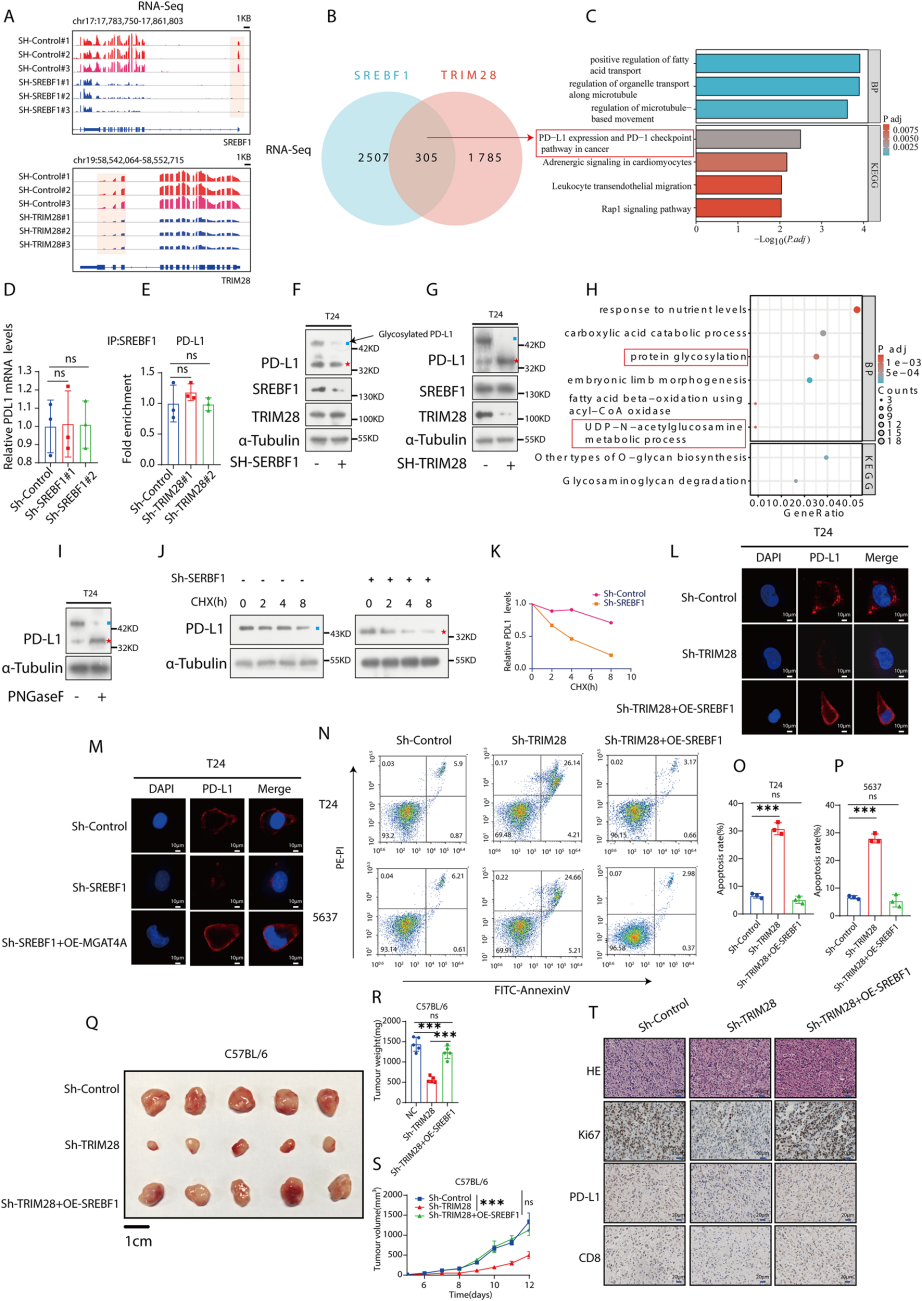
TRIM28 and SREBF1 co-regulate PD-L1 glycosylation and immune evasion in bladder cancer. **A** RNA-seq analysis of T24 cells following knockdown of SREBF1 or TRIM28, identifying differentially expressed genes (fold change > 2, P < 0.05). **B**, Venn diagram showing overlap of differentially expressed genes between SREBF1 and TRIM28 knockdowns. **C** GO/KEGG enrichment analysis of differentially expressed genes, highlighting immune-regulatory pathways associated with PD-L1 regulation. **D** SREBF1 knockdown does not alter PD-L1 mRNA levels, as assessed by RT-qPCR. **E** TRIM28 knockdown does not affect SREBF1 occupancy at the PD-L1 promoter, as shown by ChIP-qPCR. **F**, **G** Knockdown of either SREBF1 or TRIM28 alters PD-L1 species’ electrophoretic mobility, suggesting changes in post-translational modification. **H** Gene-set enrichment analysis following SREBF1 knockdown implicates N-linked glycosylation in PD-L1 regulation. **I** Treatment with PNGase F shifts PD-L1 electrophoretic mobility and reduces the 43-kDa band, confirming N-linked glycosylation. **J**, **K** Glycosylated PD-L1 is more stable than non-glycosylated forms, as assessed by protein turnover assays. **L** Immunofluorescence microscopy shows that TRIM28 knockdown reduces PD-L1 localization at the plasma membrane, while SREBF1 overexpression restores membrane residency. M, MGAT4A is required for SREBF1-driven PD-L1 glycosylation and membrane localization. **N**–**P** Co-culture assays show that TRIM28 knockdown enhances CD8 + T-cell-mediated apoptosis, which is reversed by SREBF1 overexpression. **Q**–**S** In vivo MB49 xenograft growth in immunocompetent C57BL/6 mice: TRIM28 knockdown significantly impairs tumor growth, which is rescued by SREBF1 overexpression. **T** Immunohistochemistry reveals reduced Ki67 and PD-L1 expression, with increased CD8 + T-cell infiltration, following TRIM28 knockdown, all of which are reversed by SREBF1 overexpression. Data are mean ± s.d. where applicable; two-sided statistical tests were used. *P* values: ns, not significant; *, *P* < 0.05; **, *P* < 0.01; ***, *P* < 0.001.

To define how TRIM28 cooperates with SREBF1 in BCa, we individually knocked down SREBF1 or TRIM28 in T24 cells and performed RNA-seq (Fig. 6A). Differentially expressed genes (fold change > 2 and P < 0.05) were intersected (Fig. 6B) and subjected to GO/KEGG enrichment (Fig. 6C). Beyond the canonical lipid metabolic programs linked to SREBF1, immune-regulatory pathways were significantly enriched, implicating both factors in PD-L1 control. Because SREBF1 did not alter in vitro proliferation of BCa cells, we hypothesized that SREBF1-driven effects on tumor growth depend on the tumor immune microenvironment. We next assessed PD-L1 transcription and chromatin binding. SREBF1 knockdown left PD-L1 mRNA unchanged (Fig. 6D), and TRIM28 knockdown did not affect SREBF1 occupancy at the PD-L1 promoter by ChIP-qPCR (Fig. 6E), arguing against a TRIM28-dependent, direct transcriptional mechanism. We therefore focused on protein-level regulation. Notably, knockdown of either SREBF1 or TRIM28 altered higher-molecular-weight PD-L1 species in T24 cells (Fig. 6F and G), consistent with changes in post-translational modification such as glycosylation. Gene-set enrichment following SREBF1 knockdown further implicated protein glycosylation, particularly N-linked glycosylation (Fig. 6H). It shows the differentially expressed genes in the protein glycosylation pathway (Fig. S2A). Functionally, PNGase F treatment shifted PD-L1 electrophoretic mobility and markedly reduced the 43-kDa band (Fig. 6I), supporting N-linked glycosylation of PD-L1 under the TRIM28/SREBF1 axis. We then compared the stability of glycosylated versus non-glycosylated PD-L1. Glycosylated PD-L1 was substantially more stable (Fig. 6J, K), indicating that TRIM28/SREBF1-mediated glycosylation elevates total PD-L1 by limiting protein turnover. Immunofluorescence showed that TRIM28 knockdown reduced PD-L1 localization at the plasma membrane, whereas SREBF1 overexpression partially or robustly restored membrane residency (Fig. 6L), suggesting that glycosylation promotes PD-L1 membrane localization. To delineate the mechanism promoting glycosylation, we examined genes in the “protein glycosylation” pathway and identified MGAT4A as significantly downregulated after knockdown of either SREBF1 or TRIM28. MGAT4A encodes β-1,4-N-acetylglucosaminyltransferase, which mediates N-glycan branching [24]. Mechanistically, TRIM28 enhances the enrichment of SREBF1 at the MGAT4A promoter, while showing no significant effect on another glycosyltransferase, MGAT1, or on several classical lipid metabolism enzymes, including FASN, SCD1, and ACACA. These results suggest that TRIM28 may specifically enhance the transcriptional regulation of MGAT4A by SREBF1. (Fig. S2B-F). Functionally, MGAT4A was required for SREBF1-driven PD-L1 glycosylation and membrane localization (Fig. 6M). In the TCGA-BCa cohort, higher SREBF1 and TRIM28 expression correlated with reduced immune cell infiltration (Fig. S3A), suggesting immunosuppressive activity in vivo. We further stratified bladder cancer into Basal/Squamous and Luminal subtypes and evaluated the infiltration levels of CD8⁺T cells in tumors with different expression levels of SREBF1 and TRIM28. The results showed that CD8⁺T-cell infiltration was significantly reduced in bladder cancers with high expression of TRIM28 or SREBF1 (Fig. S3B-E). Subsequently, we performed immunohistochemical staining on tumor tissues from 55 patients with bladder cancer, which further confirmed that bladder cancer tissues with high SREBF1 expression exhibited reduced infiltration of CD8⁺T cells (Fig. S4A-C). In co-culture, activated CD8 + T cells (CD3/CD28) induced greater apoptosis in tumor cells upon TRIM28 knockdown; this effect was reversed by SREBF1 overexpression in T24 or 5637 cells (Fig. 6N-P), consistent with PD-L1–mediated suppression of CD8 + T-cell cytotoxicity. In vivo, SREBF1 knockdown did not affect MB49 xenograft growth in BALB/c nude mice (Fig. S5A-C), consistent with minimal cell-intrinsic effects on proliferation. By contrast, in immunocompetent C57BL/6 mice, TRIM28 knockdown significantly impaired MB49 tumor growth, which was rescued by SREBF1 overexpression (Fig. 6Q–S). Immunohistochemistry further showed reduced Ki67 and PD-L1, together with increased CD8 + T-cell infiltration, upon TRIM28 knockdown, all of which were reversed by SREBF1 overexpression (Fig. 6T).

Collectively, these data support a model in which TRIM28 acts as a co-regulator to enhance SREBF1 occupancy at the MGAT4A promoter, thereby upregulating MGAT4A and promoting N-linked glycosylation of PD-L1. This modification stabilizes PD-L1 and facilitates its membrane localization, blunting CD8 + T-cell cytotoxicity and promoting tumor growth in immunocompetent hosts.

### Combined SREBF1 targeting and anti-PD-1 synergise in PDX

**Fig. 7:**
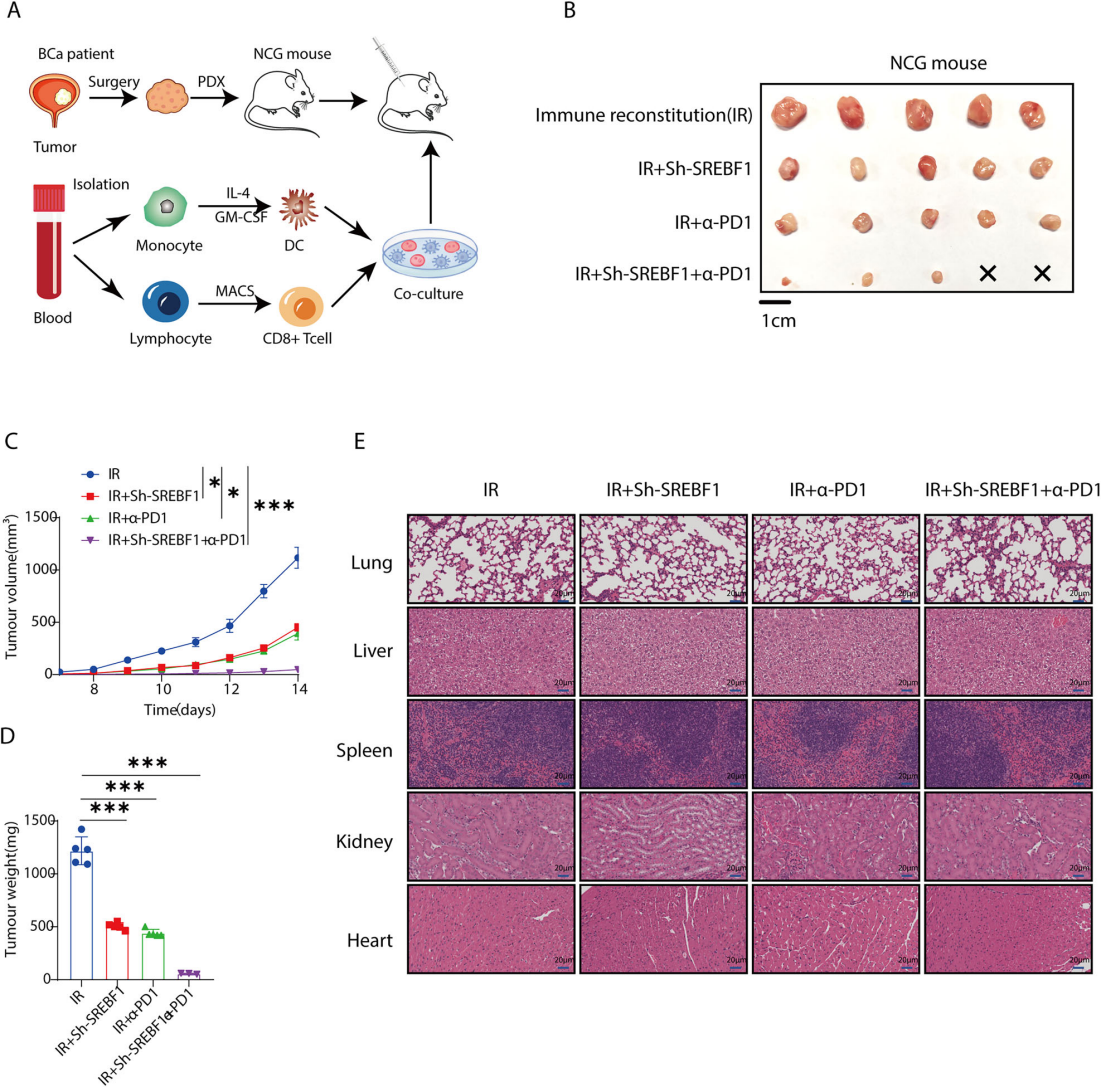
Immune augmentation and combination therapy enhance antitumor efficacy in a patient-derived xenograft (PDX) model. **A** Schematic representation of the immune augmentation-tumor co-engraftment model in highly immunodeficient NCG mice. Monocytes were isolated from human peripheral blood and differentiated into macrophages in vitro; these were subsequently co-cultured with CD8⁺ T cells, and the two cell types were then mixed and co-injected subcutaneously to partially reconstitute the human immune microenvironment. PDX fragments of bladder cancer were also implanted subcutaneously. **B**–**D** Tumor growth curves showing that both SREBF1 knockdown (sh-SREBF1) and anti-PD-1 monotherapy enhance CD8 + T-cell effector function and suppress tumor growth. The combination of sh-SREBF1 and anti-PD-1 antibody results in the most pronounced antitumor activity, significantly reducing tumor burden compared to either monotherapy. **E** Hematoxylin and eosin (H&E) staining of major organs (heart, liver, spleen, lung, and kidney) reveals no overt histopathological abnormalities or increased inflammatory infiltration in any treatment group, indicating no significant toxicity. Data are mean ± s.d. where applicable; two-sided statistical tests were used. P values: ns, not significant; *, *P* < 0.05; **, *P* < 0.01; ***, *P* < 0.001.

**Fig. 8:**
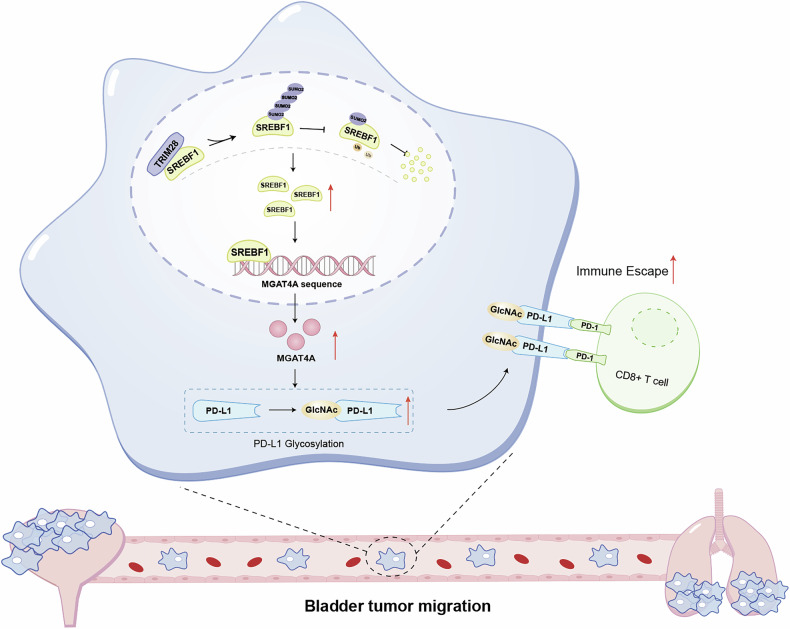
TRIM28 -SREBF1 -MGAT4A axis promotes PD-L1 N-glycosylation and immune evasion in bladder cancer. Schematic model illustrating that TRIM28 interacts with and mediates SUMO2 conjugation of SREBF1 at K470, antagonizing its K48-linked ubiquitination and stabilizing SREBF1. Stabilized SREBF1 enhances transcription of MGAT4A, leading to increased N-linked glycosylation of PD-L1, which promotes PD-L1 stabilization and plasma membrane localization, thereby suppressing CD8+ T-cell cytotoxicity and facilitating tumor immune escape.

In highly immunodeficient NCG mice, we established an immune augmentation-tumour co-engraftment model. CD8 + T cells and monocytes were isolated from human peripheral blood; monocytes were differentiated in vitro into macrophages and briefly co-cultured with CD8 + T cells before co-injection subcutaneously to partially reconstitute a human immune microenvironment. In parallel, patient-derived bladder tumour xenograft (PDX) fragments were implanted subcutaneously to model the clinical tumour milieu (Fig. 7A). After tumour establishment, animals were randomly assigned to receive a lentiviral vector delivering shRNA targeting SREBF1, an anti-PD-1 antibody, or the combination, with appropriate controls. Both SREBF1 knockdown (sh-SREBF1) and anti-PD-1 monotherapy enhanced CD8 + T-cell effector function and suppressed tumour growth; the combination produced the most pronounced antitumour activity, further reducing tumour burden relative to either monotherapy (Fig. 7B-D). To assess safety, haematoxylin and eosin staining of major organs (heart, liver, spleen, lung, and kidney) revealed no overt histopathological abnormalities or increased inflammatory infiltration across groups (Fig. 7E). Figure 8 shows the schematic diagram of the mechanism in this study.

## Discussion

SREBF1 is a well-established master regulator of de novo lipogenesis linked to invasion, metastasis, and therapeutic resistance across cancers, including urothelial carcinoma [21, 25, 26]. Consistent with this literature, we observe sustained SREBF1 overexpression in tumours, associated with adverse clinicopathological features and inferior survival. Notably, under nutrient-replete conditions, SREBF1 is dispensable for proliferative expansion but is required for migration, invasion, and pulmonary colonisation. Emphasising metabolic context reconciles reports of SREBF1‑dependent proliferation: its impact on the cell cycle is accentuated when lipid supply is limited, mTORC1/SCAP dynamics restrict nuclear SREBF1, or FASN/ACACA constitute bottlenecks [27–29]. Conversely, when exogenous lipids are abundant, SREBF1’s dominant influence may shift to membrane remodelling, cytoskeletal plasticity, and focal adhesion dynamics that drive motility [30, 31]. Testing SREBF1 dependence under lipid‑depleted or SCAP‑perturbed conditions should resolve these context‑dependent phenotypes.

We identify TRIM28 as a direct, nuclear, domain‑specific SUMO E3 ligase for SREBF1: TRIM28 B‑box1 engages the SREBF1 transactivation domain to catalyse SUMO2 conjugation at K470. This aligns with TRIM28’s co‑regulatory role and reported SUMO E3 activity via its PHD–bromodomain scaffold [17, 32]. SUMOylation at K470 antagonises K48‑linked polyubiquitin and stabilises SREBF1, consistent with paradigms in which SUMO marks shield degrons, block E3 docking, or recruit deubiquitinases to restrain proteasomal turnover. Orthogonal perturbations—including site‑directed mutagenesis, SUMO‑pathway inhibition, and TRIM28 loss/gain—converge on this model. This mechanism extends prior regulation of SREBF1 by phosphorylation‑dependent ubiquitination (e.g., the GSK3β–FBXW7 axis) and mTORC1–INSIG–SCAP trafficking [33–35], positioning SUMOylation as a complementary post‑translational layer that tunes SREBF1 protein lifespan downstream of chromatin co‑regulation. Although some studies ascribe to TRIM28 a capacity to facilitate ubiquitination of select substrates, our data indicate substrate‑ and PTM‑context specificity: at SREBF1 K470, SUMO2 rewires competition for K48‑linked chains rather than globally suppressing ubiquitination. Meanwhile, TRIM28 has also been reported to be associated with tumor immune regulation in multiple cancer types [17, 32].

Beyond lipid anabolism, we link SREBF1 to immune evasion through PD‑L1 protein maturation. Depletion of SREBF1 or TRIM28 leaves PD‑L1 mRNA unchanged yet reduces higher‑molecular‑weight species that collapse upon PNGase F treatment, indicating control at the level of N‑linked glycosylation. This accords with reports that PD‑L1 glycosylation enhances stability, engages galectins, and increases membrane residency to sustain T‑cell suppression [19, 36]. We further place MGAT4A as a convergent node downstream of TRIM28–SREBF1. Whereas earlier work in other tumour types emphasised STT3‑mediated transfer, B3GNT3‑driven poly‑LacNAc extension or MGAT5‑dependent β1,6‑branching, our data indicate that, in bladder cancer, MGAT4A‑driven tri‑/tetra‑antennary branching is rate‑limiting for PD‑L1 maturation, stability and surface retention. This divergence likely reflects tissue‑specific enzyme expression, substrate pools (e.g., UDP‑GlcNAc), and pathway bottlenecks. High‑resolution glycomics and site‑specific PD‑L1 glycopeptide mapping will clarify how MGAT4A‑dependent branching relates to MGAT5/B3GNT3 architectures across contexts.

Oncogenic and inflammatory pathways (e.g., JAK–STAT/IFNγ, EGFR/MAPK, PTEN/PI3K) robustly induce PD‑L1 transcription [37–39]. In contrast, our results nominate a glycosylation‑centric, post‑transcriptional axis as dominant in bladder cancer: perturbing SREBF1 or TRIM28 minimally affects PD‑L1 mRNA yet markedly reduces glycosylated PD‑L1 and its membrane localisation. These modalities are not mutually exclusive. We propose that transcription sets PD‑L1 supply, while glycosylation and galectin‑mediated lattice formation determine protein quality control, residence time, and checkpoint potency at the membrane. Stratifying tumours by interferon signatures, lipid‑metabolism activity, and glycosyltransferase expression may explain why PD‑L1 mRNA alone poorly predicts response in subsets of patients and motivate glyco‑PD‑L1 or branching signatures as more functional biomarkers. At a metabolic interface, our findings conceptually link lipogenesis to N‑glycan branching. SREBF1 enhances MGAT4A transcription, and TRIM28 increases SREBF1 occupancy at the MGAT4A promoter. In parallel, lipogenesis intersects the hexosamine biosynthetic pathway to elevate UDP‑GlcNAc, potentially increasing branching capacity [40]. Although we did not directly quantify nucleotide‑sugar pools, this coupling provides a unifying framework: activation of the lipid programme co‑optimises membrane biogenesis and glycoprotein maturation to sustain immune escape. Metabolomic profiling of UDP‑GlcNAc and tracer studies, together with O‑GlcNAc controls, could disentangle transcriptional versus substrate‑availability contributions to glycosylation flux.

Clinically, SREBF1 associates with muscle invasion, nodal/distant spread, higher grade, and poorer survival, supporting its potential for risk stratification. Beyond single‑analyte measures, a composite panel integrating SREBF1, MGAT4A, and glycosylated PD‑L1 (e.g., via glyco‑specific antibodies or lectin‑binding readouts) may better capture checkpoint competence than PD‑L1 IHC alone. Given intratumoural heterogeneity, spatial proteomics and multiplexed imaging could resolve niche‑level co‑localisation of SREBF1‑high lipogenic zones with glyco‑PD‑L1‑rich regions and reduced CD8+ infiltration. Points of tension and alternative explanations merit consideration. Some studies emphasise predominantly transcriptional repression roles for TRIM28, or even pro‑ubiquitin functions on specific substrates [41, 42]. Our data instead support a substrate‑ and context‑dependent SUMO E3 role on nuclear SREBF1, in keeping with chromatin co‑regulatory paradigms. Another possibility is that reduced PD‑L1 glycoforms reflect generalised ER stress or impaired protein folding. We did not observe dominant unfolded‑protein‑response transcriptional signatures; moreover, MGAT4A dependency and SREBF1 rescue favour a directed branching mechanism rather than global glycoproteostasis collapse. Nonetheless, controlling for ER‑stress markers, endocytosis rates, and PD‑L1 recycling, and testing whether exogenous galectin blockade phenocopies the effects, would further exclude indirect routes.

The immune-enhanced PDX model used in this study introduces human immune components into conventional patient-derived xenografts, allowing the investigation of tumor–immune interactions within a patient-derived tumor context. Compared with classical PDX models established in immunodeficient mice, this approach better captures functional crosstalk between tumor cells and human immune cells. Although the immune compartment is reconstructed through exogenous human immune cell transfer rather than a fully developed human immune system, the model still provides a biologically relevant platform for evaluating tumor–immune interactions. In addition, while human stromal components within the implanted tumor fragments may gradually be replaced by murine stromal cells during tumor growth, early tumor architecture and heterogeneity are preserved, and key tumor–immune interactions remain intact. Therefore, despite these limitations, the immune-enhanced PDX model represents a practical and informative system for investigating immune-related mechanisms and therapeutic responses in bladder cancer.

Our clinical analyses are retrospective; prospective validation and multi‑omic integration, including spatial proteomics, will improve biomarker performance and define beneficiary subsets. Mechanistically, structural studies around K470 and proximity labelling of SUMO/ubiquitin interactors could resolve the interface and identify cooperating PTMs. CRISPR knock‑in of K470R at the endogenous locus would strengthen causality. Glycomics (LC–MS/MS) and site‑specific PD‑L1 glycopeptide mapping should define MGAT4A‑dependent structures and dynamics. Functionally, assessing antigen presentation, additional checkpoints and myeloid programmes will clarify the breadth of this axis. In vivo, studies in fully immunocompetent models using clinically tractable SREBF1 or MGAT‑pathway inhibitors are needed to support translation and safety.

## Method

### Patients and specimens

All human BCa tissues were obtained from the Department of Urology, Fujian Medical University Union Hospital, China(Approval number:2024KJCX125). The study protocol was approved by the Fujian Medical University Union Hospital. Tumor tissues were stored at −80°C and used exclusively for protein immunoblot analyses.

### Cell lines

T24 (CL-0227) and 5637 (CL-0002) cells were purchased from Wuhan Procell Life Science & Technology Co., Ltd. (China), MB49 cells (FH1136) from Shanghai Fuheng Biotechnology (China), and HEK293T cells (#SCSP-502) from the National Collection of Authenticated Cell Cultures (Beijing, China). All cell lines were confirmed to be free of contamination. Cells were maintained in RPMI-1640 or DMEM supplemented with 10% fetal bovine serum (FBS), 100 U/mL penicillin, and 0.1 mg/mL streptomycin at 37 °C in a humidified 5% CO2 incubator. Mycoplasma contamination was routinely monitored by PCR, and cell line identity was verified by short tandem repeat (STR) profiling. Authentication and quality control were performed by the National Infrastructure of Cell Line Resource, China.

### Lentiviral shRNA

Lentiviral particles for shRNA-mediated knockdown and overexpression vectors used for in vitro studies were constructed, validated, and supplied by Bioyard Biotechnology (China). Target sequences are listed in Supplementary Table 1.

### Antibodies

All antibodies used in this study are provided in Supplementary Table 2.

### Western blotting

For protein extraction, biological triplicates were lysed in prechilled RIPA buffer containing protease and phosphatase inhibitors (Yeason, China; Cat# 20115ES60). Protein concentration was determined using a BCA Protein Assay Kit (Yeason, China; Cat# 20201ES76). Samples were resuspended in Laemmli loading buffer, sonicated (15 cycles of 30 s with intermittent cooling), denatured by boiling with β-mercaptoethanol and bromophenol blue, and subjected to SDS–PAGE. Proteins were transferred to PVDF membranes and blocked using EpiZyme rapid blocking buffer. Membranes were incubated with primary antibodies at 4°C overnight (blocking buffer containing 0.2% Tween-20). Afterward, membranes were either re-blocked for 10–15 min at room temperature in rapid blocking buffer or directly incubated with HRP-conjugated secondary antibodies for 1 h at room temperature in diluent containing 0.2% Tween-20 and 0.01% SDS. Signals were developed by enhanced chemiluminescence (ECL), visualized on X-ray film, processed in a darkroom, and analyzed by scanning.

### Cell proliferation and colony formation

EdU incorporation assays were performed according to the manufacturer’s protocol. Cells were incubated with EdU, fixed, stained, and EdU-positive cells were quantified. For colony formation, cells were seeded at low density in six-well plates and cultured for 10–14 days, then fixed, stained, and colonies were counted.

### Migration and invasion assays

For Transwell migration, cells were seeded in serum-free medium in the upper chamber, and medium containing chemoattractant was placed in the lower chamber; migrated cells were fixed, stained, and quantified. For Matrigel invasion, inserts were pre-coated with Matrigel and processed as in migration assays. For scratch-wound healing assays, confluent monolayers were scratched, debris was removed, and wound closure was imaged over time and quantified.

### Isolation of PBMCs and CD8 + T cells

Peripheral blood mononuclear cells (PBMCs) were isolated from healthy donors by Ficoll-Paque density gradient centrifugation (GE Healthcare, Cat# 17-5442-02), followed by two PBS washes to remove platelets and impurities. CD8 + T cells were purified using a CD8 + T Cell Isolation Kit (Miltenyi Biotec, Cat# 130-096-495). Purity (>95%) was confirmed by flow cytometry.

### Co-immunoprecipitation (Co-IP) and mass spectrometry

Cells transfected with the indicated plasmids were lysed in Beyotime cell lysis buffer supplemented with a protease inhibitor cocktail (CoWin Biosciences). Ten percent of the lysate was reserved as input and stored at −20 °C. The remaining lysate was incubated with specific antibodies and protein A/G magnetic beads (Thermo Scientific) at 4 °C with rotation overnight. Beads were washed five times (5 min each) with cold wash buffer, resuspended in 1× SDS loading buffer, and denatured by heating. Immunoprecipitates were stored at −20 °C or analyzed by SDS–PAGE, silver staining, or LC–MS/MS. Mass spectrometry was performed by Shenzhen Huinuobaisi Biotechnology Co., Ltd. (Shenzhen, China). Protein identification and quantification were conducted using Proteome Discoverer software (Thermo Scientific, v1.4).

### Immunofluorescence

Cells were seeded onto confocal dishes and cultured for 24 h. Cells were fixed with 4% paraformaldehyde (Beyotime) for 15 min at room temperature and permeabilized with 0.5% Triton X-100 for 15 min. After blocking with 5% bovine serum albumin (BSA; Sigma-Aldrich) for 1 h at room temperature, cells were incubated with primary antibodies at 4 °C overnight. The next day, cells were incubated with fluorescent secondary antibodies (Alexa Fluor 488 or Cy3) for 1 h protected from light. Nuclei were counterstained with DAPI when indicated. Images were acquired on a Zeiss confocal laser scanning microscope.

### Immunohistochemistry

Paraffin-embedded BCa sections were deparaffinized, rehydrated, and subjected to antigen retrieval in citrate buffer (ZLI-9079, ZSGB-BIO, China). Endogenous peroxidase activity was quenched with PV-6001 (ZSGB-BIO), followed by blocking of nonspecific binding with ZLI-9056 (ZSGB-BIO). Sections were incubated with primary antibodies and developed using an HRP detection kit (ZLI-9017, ZSGB-BIO) according to the manufacturer’s instructions. Two experienced pathologists independently evaluated the staining. The staining index (SI) was calculated based on staining intensity and the percentage of positive cells. Intensity was scored as 0 (negative), 1 (weak), 2 (moderate), or 3 (strong).

### Animal studies

Male BALB/c, NCG, and Balb/c mice (6–8 weeks old) were purchased from Beijing Vital River Laboratory Animal Technology Co., Ltd. (Charles River, Beijing, China). All animal procedures were approved by the Institutional Animal Care and Use Committee (approval no.:IACUCFJMU 2024-Y-2274). For subcutaneous implantation, 1.0 × 10^6 MB49 cells were injected subcutaneously into the right dorsal flank of BALB/c or Balb/c male mice. Tumor volumes were measured daily with calipers and calculated as length × width^2 × 0.5. Tumor immune phenotypes were analyzed by flow cytometry. Tumors were fixed, paraffin-embedded, and stained with H&E. Statistical comparisons were performed using two-tailed Student’s t-tests.

### Patient-derived xenograft (PDX) model

For PDX establishment, fresh human bladder tumor fragments were implanted subcutaneously into NCG mice. When tumors reached 100 mm^3, mice were euthanized, and tumors were excised, cut into ~1 mm^3 pieces, and serially passaged into subsequent generations of NCG mice. A stable BCa PDX line was established after three passages. For adoptive T-cell transfer, tumor-specific activated CD8 + T cells and dendritic cells (DCs) were administered via tail vein to reconstitute a humanized immune system. Tumor burden was assessed weekly by palpation and caliper measurement; tumor volume (mm^3) = (length × width^2)/2. Mice were euthanized when tumors reached 1,500 mm^3 or when ulceration occurred.

To generate an immune-reconstituted PDX system, PBMCs were isolated from HLA-A2+ healthy donors and differentiated into DCs in VIVO medium (04-418Q, Lonza) supplemented with 100 ng/mL GM-CSF and 30 ng/mL IL-4 (PeproTech), with medium and cytokines refreshed every 3 days. On day 6, DC maturation was induced by 10 ng/mL TNF-α (PeproTech) for 24 h. Mature DCs were then pulsed for 24 h with tumor lysates from HLA-A2+ patients prepared by repeated freeze–thaw cycles in liquid nitrogen to enhance antigen presentation, and used for subsequent immunotherapy. Tumor-specific CD8 + T cells were generated by isolating CD8 + T cells from the same donor and co-culturing them with mature DCs at a 5:1 ratio in VIVO medium (Lonza) supplemented with 25 IU/mL IL-2 (PeproTech) for 6 days. These cells were used for human immune system reconstitution or adoptive cell therapy.

### Statistics and reproducibility

All in vitro experiments were independently repeated at least three times with consistent results; representative data are shown unless otherwise indicated. In vivo experiments included at least three biological replicates per group, and independent experiments showed similar trends. Two-group comparisons used two-tailed unpaired Student’s t-tests; one-way ANOVA was applied for multiple groups with a single factor; two-way ANOVA was used for analyses involving two independent variables. Survival analyses employed the log-rank (Mantel–Cox) test. Statistical analyses were performed using GraphPad Prism (v8.0.1). Data are presented as mean ± s.e.m. unless otherwise noted. Mice were randomly assigned to treatment groups, and investigators were blinded to group allocation during experiments to minimize bias.

## Supplementary information

Figure S1 | GPS-SUMO prediction of potential SUMOylation sites on SREBF1. A, GPS-SUMO prediction algorithm was used to identify potential SUMOylation sites on SREBF1. The five candidate lysine residue

Figure S2 | TRIM28 enhances SREBF1 enrichment at the MGAT4A promoter and regulates genes involved in protein glycosylation. (A) Differentially expressed genes in the protein glycosylation pathway iden

Figure S3 | Association between TRIM28/SREBF1 expression and immune cell infiltration in bladder cancer. (A) Correlation analysis between TRIM28 or SREBF1 expression and immune cell infiltration level

Figure S4. High SREBF1 expression correlates with reduced CD8⁺ T-cell infiltration in bladder cancer tissues. (A,B) Representative immunohistochemical staining images of SREBF1 (A) and CD8 (B) in blad

Figure S5 | SREBF1 knockdown does not affect MB49 xenograft growth in BALB/c nude mice. A–C, In vivo MB49 xenograft model in BALB/c nude mice shows that SREBF1 knockdown does not significantly alter t

Supplementary Table 1: Target sequences of shRNAs and siRNA used in this study

Supplementary Table 2: Details of the antibodies used in this study.

aj-checklist

WB
