# Supplementary material for: TRIM28-mediated SUMOylation of SREBF1 drives PD‑L1 N‑glycosylation and immune evasion in bladder cancer
Source: Cell Death Dis. 2026 May 18;17(1):693. doi: 10.1038/s41419-026-08798-8 (PMC13448484; doi:10.1038/s41419-026-08798-8)

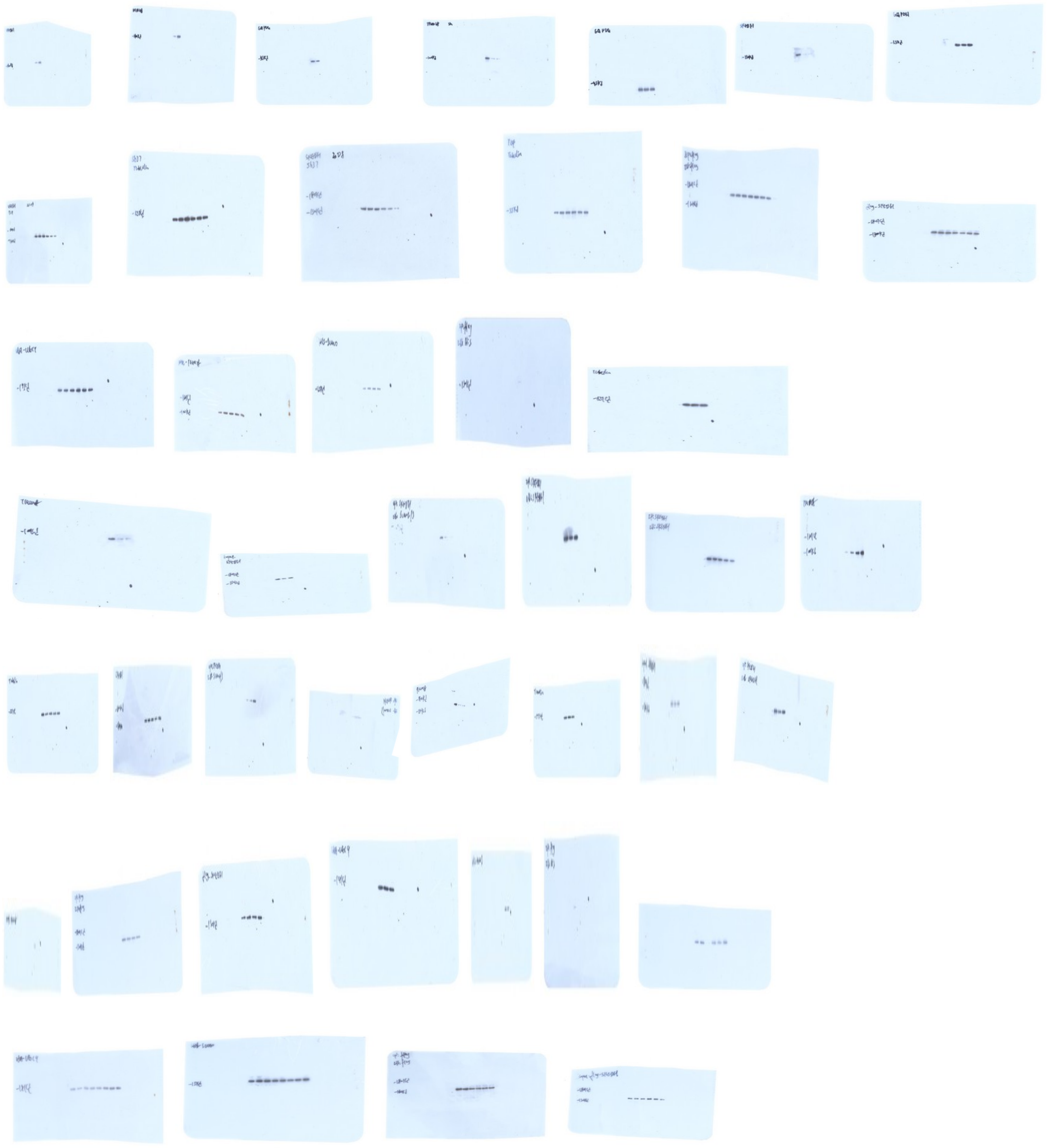

Supplement: Supplementary file 2 [file 41419_2026_8798_MOESM9_ESM.docx]
