# Supplementary material for: TRIM28-mediated SUMOylation of SREBF1 drives PD‑L1 N‑glycosylation and immune evasion in bladder cancer
Source: Cell Death Dis. 2026 May 18;17(1):693. doi: 10.1038/s41419-026-08798-8 (PMC13448484; doi:10.1038/s41419-026-08798-8)
Supplement: Supplementary file 2 [file 41419_2026_8798_MOESM6_ESM.docx]

| Supplementary Table 1: Target sequences of shRNAs and siRNA used in this study | | |
| --- | --- | --- |
| **Gene** |  | **Sequence (5’-3’)** |
| TRIM28 | SH-RNA#1 | GAGAATTATTTCATGCGTGAT |
|  | SH-RNA#2 | CTGAGACCAAACCTGTGCTTA |
| SREBF1 | SH-RNA#1 | CATCGACTACATCCGCTTCTT |
|  | SH-RNA#2 | CCCTGTGCTGACGGAAGCCAA |
