# Supplementary material for: TRIM28-mediated SUMOylation of SREBF1 drives PD‑L1 N‑glycosylation and immune evasion in bladder cancer
Source: Cell Death Dis. 2026 May 18;17(1):693. doi: 10.1038/s41419-026-08798-8 (PMC13448484; doi:10.1038/s41419-026-08798-8)
Supplement: Supplementary file 3 [file 41419_2026_8798_MOESM7_ESM.docx]

| Supplementary Table 2: Details of the antibodies used in this study. | | | |
| --- | --- | --- | --- |
| Protein | Catalog No | Source | Dilution or amount |
| TRIM28 | 15202-1-AP | Proteintech | WB:1:1000,IP/1μg,IHC/1:100 |
| SREBF1 | 82684-3-RR | Proteintech | WB:1:2000,IP/1μg,IHC/1:100 |
| Flag-tag | 80801-2-RR | Proteintech | IP/1 μg ,WB/1:2000 |
| HA-tag | AE036(AB_2771924) | ABclonal | IP/1 μg, WB/1:1000 |
| c-Myc-tag | 18583 | Cell Signaling Technology | IP/1 μg, WB/1:1000 |
| α-Tubulin | 66031-1-Ig(AB_11042766) | Proteintech | WB/1:5000 |
| GST | ab138491 | abcam | WB/1:1000 |
| PDL1 | 66248-1-Ig(AB_2756526) | Proteintech | WB/1:2000 |
| SUMO2/3 | 10699-1-AP(AB_3669122) | Proteintech | IP/1 μg,WB/1:500 |
| His-tag | ab18184 | abcam | IP/1 μg,WB/1:1000 |
